# Differences in severity and resource utilization for medical and surgical ICU patients

**DOI:** 10.1186/cc12428

**Published:** 2013-03-19

**Authors:** BH Nathanson, WT McGee, E Lederman, TL Higgins

**Affiliations:** 1 OptiStatim LLC, Longmeadow, MA, USA; 2 Baystate Medical Center, Springfield, MA, USA

## Introduction

Medical and surgical patients use the ICU differently. Resources may not always be allocated by severity of illness, but by custom or habit, particularly if different groups administer bed control and triage. Specialty-specific differences may exist even when a single team controls triage. Variability in resource utilization has important implications for cost-containment and triage.

## Methods

Patients admitted to a single, closed medical/surgical ICU with full-time intensivists and unified triage control in a large, university-affiliated hospital were evaluated during 2011 to 2012. Patients who died in the ICU were excluded. The days of discharge (D/C) and severity using APACHE IV and its related Acute Physiology Score (APS) component were calculated daily for the first 7 days. Trend was assessed across days by Cuzick's test.

## Results

**Figure 1:**
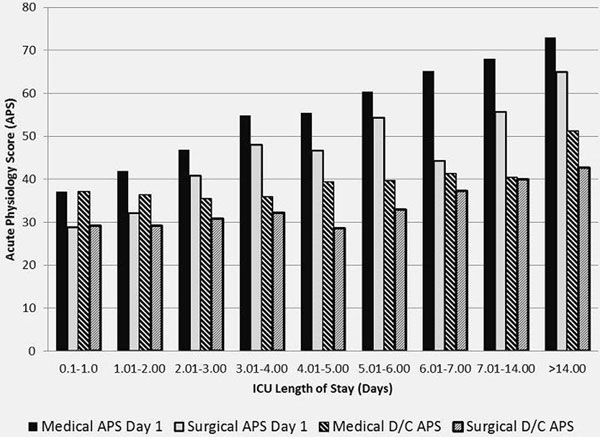


A total of 719 surgical and 925 medical patients met inclusion criteria. In total, 20.2% of surgical and 21.3% of medical patients had an ICU LOS <1; *P *= 0.58. Admission severity was correlated with length of stay, *P *= 0.014 for both medical and surgical patients. Medical patients are sicker on admission and D/C from the ICU than surgical patients (*P *0.05) (Figure 1).

## Conclusion

ICU utilization differed by patient type even with unified triage control within a single unit. Surgical patients were less severely ill on admission to and D/C from the ICU. A significant percentage of medical and surgical patients are D/C within 1 day and may be more efficiently served in a less resource-intensive environment. The reasons for the differences in ICU utilization for surgical versus medical patients require clarification and may have implications for both resource utilization and cost.

